# Are children and schools a COVID-19 threat?

**DOI:** 10.1016/j.puhip.2021.100102

**Published:** 2021-03-10

**Authors:** Andrew CK. Lee, Joanne R. Morling

**Affiliations:** The University of Sheffield, UK; The University of Nottingham, UK

The public health measures and restrictions introduced around the world to control the COVID-19 pandemic have had profound impacts on numerous aspects of life. In particular school-age children will have had their childhood blighted, not least by the disruption to their education arising from school closures. As a pandemic control measure, the latter is a contentious issue in many countries with proponents for and against school closures. But the issue is far more complex and nuanced, and it is a false dichotomy to judge school settings simply as ‘safe’ or ‘not safe’ when true infection risk is a spectrum.

Some of the proponents of school closure may feel that children are likely “vectors” of infection and that schools are therefore high-risk settings for the spread of infection. There is understandably a lot of public concern especially when infection rates in the community are high, and the intuitive reaction is to shut schools to protect children, teachers and the wider community. But intuition does not equate to evidence and public perception of risk may not be accurate especially if there is a significant overlay of heightened concern. With the emergence of new variants, such as the B.1.1.7 variant in the UK, this has led to public clamour to shut schools. However, current evidence indicates children are no more susceptible to these variants than adults, and experts caution against school closures on the basis of incomplete information [1]. Neither is it known for certain how effective school closures are as a pandemic measure [2].

So what do we know so far about the actual risk of infection posed by children? There have been several reviews of the evidence by various governments and organisations around the world, including the WHO [3], the UK Royal College of Paediatrics and Child Health [4], and the European Centre for Disease Control and Prevention [5]. Thus far the evidence suggests that children tend to be less susceptible to SARS-CoV-2 infection and experience less severe illness [6]. They are more likely to have milder symptoms, shed viral RNA for less time, and the duration of illness in children is probably shorter. Consequently, children are less likely to account for onward transmission or be superspreaders of infection.

Whilst the evidence currently indicates children are not a key driver of the epidemic, it is not disputed that children can be infected and can transmit infections. However, the susceptibility to being infected does not necessarily equate to the propensity to infect others. So whilst young children can get infected they are not as infectious as older teenagers, for example, whose infectivity is more similar to adults.

Disentangling the actual contribution of children to infections is not easy. One modelling study from Wuhan suggests children may be more infectious than adults in the household setting [7]. However, there remain questions as to how direction of transmission can be elucidated from population studies conclusively, especially as infections tend to be introduced into household settings by other adults [8]. Similarly, outbreak cases in schools are more likely to be introduced by adults, and transmission is usually staff-to-staff or staff-to-student rather than between students or from students [9].

Another issue is the problem that ‘outbreaks’ in schools may not be true outbreaks with evidence of in-school transmission of infection. Instead, they may be clusters of cases associated with the school setting but the infections were acquired in the community, and most likely from household transmission where attack rates are highest. School-related clusters are not proof of transmission in the school setting [5]. Indeed, schools tend to comprise only a minority of settings for transmission. With appropriate precautions and infection control measures, school settings can be made relatively safe. Indeed, one US study found school transmissions of infection were considerably less than infection rates in the wider community [10].

Moreover, infection levels in children and schools will reflect the situation in the wider community. Rather than children and schools being seen as a risk to the community, the converse also holds true – i.e. what is the risk to children and schools posed by the community? There is a strong association with community transmission with the risk of outbreaks in school rising by 72% when the community incidence increases by 5 per 100,000 population [5].

There are also concerns as to whether school staff are at greater risk of infection. Data from the UK found teachers were not at increased risk of infection compared to other occupational groups [11]. Likewise, investigations from Germany, France, Ireland, Australia, Singapore and the US found no or very low secondary attack rates within school settings [5]. This suggests public health measures implemented in schools can and do work.

School closures adversely affect learning opportunities for children which could have long-term, detrimental effects. It is not easy to deliver remote learning opportunities to all children. In the UK, it has been reported that a third of pupils do not have access to an internet-enabled device [12]. The loss of learning may also widen social inequalities and exacerbate entrenched inequalities by disproportionately harming marginalised and vulnerable children and families [13]. For example, private school pupils are more likely to have more learning time than state school pupils. It is also more difficult for low-income families to do home schooling. There are also gender inequalities as working mothers tend to be disproportionately affected [14].

School closures and home isolation of children are also associated with increasing mental ill health and poor wellbeing of children, domestic abuse, and neglect [15]. Children with disabilities are especially affected as they are more isolated than others and the support services they need may be closed or limited in provision. There are also consequences for parents and carers, who may experience greater stress, adverse impacts on their job security and impacts on their mental health.

School closures may be necessary when infection levels in the population are very high and should only really be implemented as a last resort as part of wider pandemic control measures. They should not be undertaken lightly due to the considerable consequences posed. There are also other important considerations such as whether schools can operate safely and effectively, especially if staff are off sick or in isolation, how children can get to and from schools, and what impact opening schools has on parents and carers mixing.

The evidence so far tends to indicate that children and schools are ‘lower risk’ rather than ‘zero risk’ for infection. That said the evidence base is still growing and there remains a need for more robust studies. Public health practitioners will need to keep abreast with the evolving evidence base and interpret it for their local contexts. They may need to tackle growing public anxiety and communicate a measured view to the public and policymakers in this febrile environment. In deciding what public health measures to implement, it is ultimately a matter of balancing risks and benefits. There may be no right answers, only least worst options.
